# The Relaxation Exercise and Social Support Trial (RESST): a community-based randomized controlled trial to alleviate medically unexplained vaginal discharge symptoms

**DOI:** 10.1186/1471-244X-12-195

**Published:** 2012-11-09

**Authors:** Loulou Kobeissi, Ziyad Mahfoud, Brigitte Khoury, Fayssal El Kak, Zeina Ghantous, Marwan Khawaja, Rima Nakkash, Sami Ramia, Huda Zurayk, Ricardo Araya, Tim J Peters

**Affiliations:** 1 Epidemiology and Biostatistics Division, Center for Middle Eastern Studies, University of Arizona, Tucson, Arizona, USA; 2 Center for Research on Population and Health Epidemiology and Population Health Department Faculty of Health Sciences, American University of Beirut, Beirut, Lebanon; 3 Department of Public Health, Weill Cornell Medical College, Doha, Qatar; 4 Department of Psychiatry-Faculty of Medicine, American University of Beirut, Beirut, Lebanon; 5 Department of Health Promotion and Community Health Faculty of Health Sciences, American University of Beirut, Beirut, Lebanon; 6 Center for Research on Population and Health Epidemiology and Population Health Department Faculty of Health Sciences, American University of Beirut, Beirut, Lebanon; 7 Center for Research on Population and Health Department of Health Promotion and Community Health Faculty of Health Sciences, American University of Beirut, Beirut, Lebanon; 8 Medical Lab Sciences Program Faculty of Health Sciences, American University of Beirut, Beirut, Lebanon; 9 Academic Unit of Psychiatry School of Social and Community Medicine, University of Bristol, Bristol, UK; 10 School of Clinical Sciences, University of Bristol, Bristol, UK

## Abstract

**Background:**

Symptoms such as medically unexplained vaginal discharge (MUVD) are common and bothersome, leading to potentially unnecessary use of resources.

**Methods:**

A community-based individually randomized controlled trial to assess the effectiveness of a relatively simple, culturally appropriate multi-component intervention on reducing reported MUVD, among women suffering from low-moderate levels of common mental distress. The setting was a socio-economically deprived, informal settlement in the southern suburbs of Beirut, Lebanon. The intervention comprised up to 12 group sessions implemented over a six-week period, each divided into a psychosocial and a relaxation exercise component. The primary outcome was self-reported MUVD, which was defined as a complaint of vaginal discharge upon ruling out reproductive tract infections (RTIs), through lab analysis. Anxiety and/or depression symptoms were the secondary outcomes for this trial. These were assessed using an Arabic validated version of the Hopkins Symptoms Checklist-25 (HSCL-25). Assessments were done at baseline and six months using face-to face interviews, pelvic examinations and laboratory tests. Women were randomized into either intervention or control group. Blinding on the intervention status was not possible for both logistic and ethical reasons, especially as knowledge of involvement in the intervention was integral to its delivery. Intent to treat analysis was used.

**Results:**

Of 75 women randomized to the intervention, 48% reported MUVD at 6 months compared with 63% of 73 in the control group (difference of -15%, 95% confidence interval (CI) -31%, 0%, p=0.067). Adjustments for baseline imbalances and any factors relating to consent had no appreciable effect on these results. The risk of MUVD was reduced in absolute terms by 2.4% for each intervention session attended (95% CI -4.9%, 0.0%, p=0.049). While there was also marginal evidence of a beneficial effect on anxiety, there was no evidence of mediation of the effect on MUVD through measures of common mental disorders.

**Conclusion:**

This study confirms that MUVD is an important public health problem. While the benefits of this intervention may appear modest, the intervention offers an opportunity for women to enhance their problem-solving skills as well as use physical relaxation techniques that can help them deal with stressful in their lives. Further research is needed in a variety of contexts, for different populations and preferably involving larger randomized trials of such an intervention.

**Trial registration:**

* Title of trial: The Relaxation Exercise and Social Support Trial

ISRCTN assigned: ISRCTN98441241

Date of assignation: 10/09/2010

Link: http://www.controlled-trials.com/ISRCTN98441241

* Also registered at the Wellcome Trust register:

http://www.controlled-trials.com/mrct/trial/469943/98441241

## Background

Unexplained somatic symptoms are a frequent reason for consulting health professionals. Repeated consultations and potentially unnecessary and often expensive diagnostic procedures add to the burden of these complaints [1]. Common symptoms for which there is no evident medical explanation are: pain in the limbs, dyspnea, headache, back pain, coughing, and vaginal discharge.

In many parts of the world, vaginal discharge is among the most commonly reported and bothersome complaints by women in their reproductive years [2-4]. In Muslim communities, vaginal discharge is considered troublesome because it affects the woman’s prayer requirements to be “clean” [5]. Vaginal discharge can be a consequence of reproductive tract infections (RTIs) [6-9]; but this is not always the case [10,11]. For instance, in a community-based study in Egypt, 77% of interviewed women reported abnormal vaginal discharge in the preceding three months, among whom only 52% had a confirmed RTI [8].

From the Urban Health Survey in Lebanon, 38% of ever-married women aged 15-59 years in Hay el Sellom complained of vaginal discharge, of whom 71% reported being ‘bothered’ by the complaint. Of those reporting vaginal discharge, 64% had consulted a health provider or planned to do so, of whom only a minority had RTIs [7]. Another similar Lebanese study found 24.5% of the women reporting vaginal discharge, only 9.3% of whom actually suffered from RTIs [12]. Hence, this poses the following inquiry: *Could Medically unexplained vaginal discharge (MUVD) be explained by common mental disorders (CMDs) such as anxiety and depression?*[9]. Several studies have observed an association between anxiety and/or depression with medically unexplained gynaecological symptoms such as pelvic pain and abnormal vaginal discharge [6,9,13-16]. Many studies have shown that this association remains after adjusting for relevant risk factors and/or RTIs [17,18].

Available research focuses on intervention strategies to improve CMDs, and it remains to be demonstrated whether such strategies improve MUVD. This paper describes the results of a community-based randomized controlled trial to decrease the burden of MUVD by focusing on CMDs. The trial aimed to assess the effectiveness of a relatively simple, culturally appropriate multi-component intervention on reducing reported MUVD, among women suffering from low-moderate levels of common mental distress.

## Methods

### Design

An individually randomized controlled trial (RCT) [19].

### Setting

Hay el Sellom in the southern suburbs of Beirut, Lebanon, an informal settlement of approximately 150,000 mainly Lebanese Shiites, with low levels of basic health care services, education and physical infrastructure [20].

### Recruitment

A total of 33 schools, three large factories, 14 gynaecological clinics and eight satellite network providers facilitated recruitment over six weeks (1 April to 15 May 2009), administering a questionnaire to all potentially eligible women. This questionnaire comprised symptoms of vaginal discharge, general inclusion/exclusion criteria and (for those reporting vaginal discharge) items relating to common mental disorders (CMDs). To rule out RTIs, women reporting vaginal discharge were referred for laboratory tests and a pelvic examination conducted by female gynaecologists trained in the protocol. Swab specimens were transported and analyzed by a trained technician from the American University of Beirut laboratory. The following lab tests were used to rule out each of the five different RTIs of interest to this trial (coupled with their respective sensitivities and specificities): COBAS AMPLICOR Nisseria gonorrhea/Chlamydia trachomatis Test” (Roche Molecular Diagnostics) was used for qualitative in vitro detection of N. gonorrhea (sensitivity 96.4%, specificity 97.9%) and C. trachomatis (sensitivity 93.4%, specificity 96.7%). “Tv latex” and “Candida Latex” (Kalon Biological Ltd) were used for T. vaginalis (sensitivity 95%, specificity 99%) and candidiasis (sensitivity 80%, specificity 100%) detection. Nugent Score technique (sensitivity 87.5%, specificity 95%) was followed for the assessing of bacterial vaginosis [21-25].

Inclusion criteria were: currently married women aged 18-49; reporting symptoms of vaginal discharge; low to moderate scores on the Hopkins Symptom Checklist-25 (HSCL-25) (ranging between 2.1-3.3 for the depression sub-scale and 2.0-3.2 for the anxiety sub-scale); signed informed consent. Exclusion criteria were: pregnant or less than 8 weeks postpartum; post-menopause or hysterectomy; reporting treatment for severe mental illness; positive RTI test (bacterial vaginosis, trichomoniasis, fungal infection, chlamydia, and gonorrhoea).

Two different consent forms were used. The first was used during the recruitment phase to obtain permission to check for the inclusion/exclusion criteria including the pelvic examination and the lab tests. This consent form was read to the woman by an interviewer in front of a witness prior to the pelvic examination. The second consent was obtained by trained trial staff from women who were eligible to participate in the trial. It ensured that women understood the trial procedures and reasons behind their eligibility.

### Randomization, concealment of allocation and blinding

Randomization was performed using a computer-generated allocation schedule, produced by an individual not involved in recruitment. The original intention was that the baseline measures and consent were to be obtained before allocation was determined remotely (by telephone) and then revealed to the woman. In the event, logistical constraints at the recruitment centres meant that remote allocation was not feasible and allocation was performed using the computerized system as planned, but in the field rather than remotely. An unintended consequence of this change in procedure was that the women were informed of their allocation into either the intervention or the control arm before they gave final written consent. As will be detailed in the analysis section, this departure from protocol necessitates particularly extensive comparison of characteristics of the (consenting) women at baseline, with suitable control for any differential selection across the trial arms. Blinding of allocation after randomisation was not feasible given the nature of the intervention.

### Intervention

This consisted of 12 group sessions implemented over a six-week period using local facilities, given only to the intervention arm of the trial. Each session was divided into two parts (delivered in an order determined by practical circumstances): a psychosocial component and a relaxation exercise component.

The psychosocial component lasted approximately 75 minutes and was delivered by five Masters level clinical psychologists, assisted by five social workers as co-moderators. These sessions were divided into directed and semi-structured social support discussion sessions, incorporating problem-solving skills building as well as venting. Once a group was formed its members remained in the same group throughout. Those delivering the intervention received two days of training from a senior clinical psychologist, and were provided with a manual describing each session in detail.

The relaxation and exercise component involved 30 minutes sessions run by physical trainers. The exercises were introduced gradually. They included teaching women how to engage in visual guided imagery exercises on their own, coupled with stretching and progressive muscle relaxation. Each session started with a summary of the previous session, a review of what was being practiced at home, followed by introducing an additional technique. A manual was also developed by a physical fitness specialist who supervised and trained the physical trainers over two days. A brief pamphlet was given to participants describing the components of each session.

The control group was a treat later group, which received the intervention after the study was completed. Those women in the control group were divided into groups and offered the same intervention protocol as in the trial, using the same facilitators and locale. During the trial controls received usual care but they were followed up by two clinical psychologists (as requested by the IRB), over the phone, every two weeks by administering the HSCL-25 -in order to ensure that their CMD status did not regress.

### Outcome assessments

Data collection took place at baseline, 1.5 months and six months using face-to-face interviews conducted by trained interviewers. The primary outcome was MUVD at six months. A woman was considered to have MUVD if she reported a complaint of vaginal discharge as assessed by the question: “are you complaining currently from vaginal discharge?” and was concurrently not suffering from any RTIs, as confirmed by pelvic exam and laboratory tests. Further questions asked whether or not they were bothered by MUVD, to indicate the colour, odour, thickness, consistency and frequency of the discharge.

Secondary outcomes included common mental disorder (CMD) as assessed by the Hopkins Symptom Checklist 25 (HSCL-25). This is known to have good psychometric properties [26,27] but was subjected to further field-testing in Lebanon prior to the trial. From these investigations, cut-off values of 2.10 and 2.00 on the HSCL-25 were determined for depression and anxiety respectively [28].

The third secondary outcome was somatisation, using the Scale for Assessment of Somatic Symptoms. The main purpose of the 1.5 months follow-up was to maintain contact with the women and is not presented further here.

### Statistical considerations

#### Sample size

Allowing for 10% attrition, 80-102 women in each group would provide 80-90% power to detect a difference of 20 percentage points (10% vs. 30%) in the primary outcome of reported MUVD at 6 months, using a 2-sided 5% significance level. There is no previous study to inform this effect size directly, but differences in CMD recovery rates of up to 40% between usual care and active interventions for CMD have been found [8,9]. A 20 percentage point difference in MUVD was chosen because in our judgement such a difference would be clinically worthwhile and the effects on CMD suggest that such a difference is plausible.

#### Data analysis

Using SPSS version 16 and Stata version 11 the trial was analyzed and reported in accordance with CONSORT guidelines. Descriptive statistics were used to compare the two groups as randomized on 14 socio-demographic and clinical variables. Since (final) consent was obtained after randomization, those consenting and not consenting were compared on the basis of the same 14 characteristics.

The primary analysis involved calculating the difference in the percentages of women with MUVD at 6 months between the groups as randomized (including the 95% confidence interval (CI) and the number-needed-to-treat. For interpretation alongside secondary analyses the primary comparison was repeated using logistic regression to obtain the odds ratio, its 95% CI and p-value. The main secondary analyses involved multivariable logistic regression to adjust for baseline imbalances across the groups of (randomized and consented) women and those characteristics associated with consent. The regression analyses were then repeated using multiple regression models for the three secondary outcomes. All regression analyses were performed before and after imputing missing outcome data using multiple imputations by chained equations [29].

Additional secondary analyses for explanatory purposes involved investigating clustering effects of the intervention groups women were assigned to, using mixed effects regression for the primary outcome. Potential mediators were investigated by adding (separately) changes in HSCL-25 Anxiety and Depression sub-scales and the somatisation scale scores to the original regression models with the primary outcome. Adherence effects were investigated using instrumental variables regression with a linear term for the number of sessions attended. These were run both on the risk difference scale for the MUVD outcome and following a probit transformation. The results for both sets of models were very similar and only the former are reported. The final set of secondary analyses involved adding appropriate interaction terms to the regression models for the primary and secondary outcomes to investigate differential effects of the intervention according to age and baseline HSCL-25 scores, all as continuous variables.

## Results

**Figure 1:**
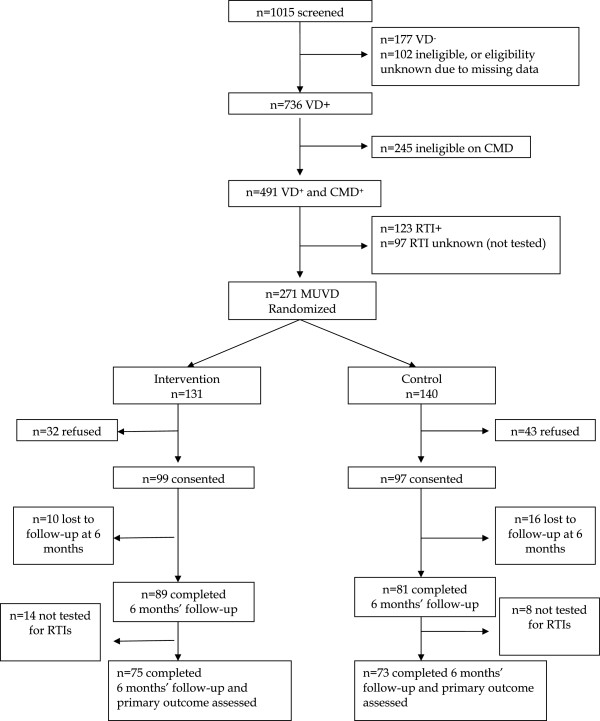
Participant flow (VD – vaginal discharge; CMD – common mental disorders; RTI – reproductive tract infection; MUVD – medically unexplained VD).

A total of 1015 women were screened over six weeks of recruitment (Figure 1). Among these, 736 (73%) reported having experienced vaginal discharge of whom 491 (67%) had symptoms of CMD (low or moderate). After excluding those testing positive for any RTI (medically explained vaginal discharge) and the 97 women who were not tested for RTIs, 271 were randomized (Figure 1). As described above, consent to participate in the trial was then sought; 32 (24%) and 43 (31%) of women in the intervention and control groups respectively refused to take part at this point. This left 99 intervention and 97 control women randomized and subsequently consenting, of whom 10 and 16 were lost to follow-up respectively. Of the remaining women, 14/89 and 8/81 tested positive for RTIs in the 6-month assessment for the intervention and control groups respectively, resulting in 75 (76% of those randomized and consenting) and 73 (75% of 97) women on whom the primary outcome was known.

Socio-demographic characteristics were similar across the study arms at baseline (Table 1), although those in the intervention arm were slightly more likely to be employed in skilled labour and have husbands with lower educational levels. Potentially more important are the higher proportions of consenting women in the intervention arm reporting bothersomeness, wetting underpants and reasons other than infection for their vaginal discharge (Table 1). While this may raise concerns about differential consent across the arms in terms of perceived problems due to vaginal discharge, there are no differences between the arms in baseline mental health scores. However, overall the 196 women who consented were more likely than the 75 women who refused consent after randomization to have never worked, to attribute their vaginal discharge to stress and/or emotional causes, and less likely to report wetting underpants due to vaginal discharge (data not shown). Moreover, those consenting had higher HSCL-25 scores for anxiety and depression (both worse by about a third of a standard deviation). In addition, at baseline, women in both arms did not differ in terms of IUD use, oral contraceptive use, or seeking medical consultation for their condition (6.5% in the intervention arm vs 10.5%; 11.8% in the intervention arm vs 9.6% in the control arm; 34% in the intervention arm vs 40.4% in the control arm, respectively). These observations indicate that the following variables warrant further consideration in the analysis: women’s type of work; husband’s educational level; bothersomeness, wetting underpants and perceived reasons for vaginal discharge; HSCL-25 anxiety and depression scores.

**Table 1:** Baseline characteristics across the arms of the trial amongst women who consented to participate (values are n (%) unless otherwise stated)

**Variable**		**Intervention n=99**	**Control n=97**
Age in years	Mean (sd)	34.3 (6.3)	34.9 (7.3)
Education			
Illiterate/Less than Elementary		25 (25.3%)	30 (30.9%)
Elementary		41 (41.4%)	40 (41.2%)
High school		20 (20.2%)	14 (14.4%)
Vocational		8 (8.1%)	8 (8.2%)
College/University		5 (5.1%)	5 (5.2%)
Type of work of the woman			
Never worked		59 (59.6%)	62 (63.9%)
Unskilled		11 (11.1%)	12 (12.4%)
Skilled labour		16 (16.2%)	8 (8.2%)
Professional		13 (13.1%)	15 (15.5%)
Education of husband			
Illiterate/Less than Elementary		40 (40.4%)	31 (32.0%)
Elementary		32 (32.3%)	42 (43.3%)
High school or above		27 (27.3%)	24 (24.7%)
Work status of husband			
Not currently working		2 (2.0%)	4 (4.1%)
Currently works		97 (98.0%)	93 (95.9%)
Type of work of husband			
Unskilled		24 (24.2%)	21 (21.6%)
Skilled labour		20 (20.2%)	16 (16.5%)
Professional		2 (2.0%)	2 (2.1%)
Company employees		26 (26.3%)	33 (34.0%)
Army/security		6 (6.1%)	6 (6.2%)
Unspecified		21 (21.2%)	19 (19.6%)
Annual family income in 1,000 LL (1US$=1,500LL)	Median(IQR)	800 (400)	750 (400)
Bothered by VD		76 (76.8%)	67 (69.8%)
Report wetting underpants due to VD		80 (81.6%)	63 (66.3%)
Woman attributes VD to:			
Stress/Emotional		14 (14.4%)	7 (7.3%)
Infection		31 (32.0%)	43 (44.8%)
Other		19 (19.6%)	12 (12.5%)
Do not know		33 (34.0%)	34 (35.4%)
Satisfied living in this area		64 (65.3%)	59 (60.8%)
Intend to leave the area		55 (56.1%)	57 (58.8%)
HSCL-25 Anxiety score	Mean (sd)	2.5 (0.4)	2.4 (0.4)
HSCL-25 Depression score	Mean (sd)	2.5 (0.4)	2.4 (0.4)

At six months, a smaller proportion in the intervention arm reported MUVD than in the control arm (Table 2). The absolute difference and the (unadjusted) odds ratio were sizeable, but there was only weak evidence of a difference beyond chance (p=0.067). From the unadjusted figures the number-needed-to-treat was just under 7. Adjustment for the variables related to consent and/or exhibiting any suggestion of baseline imbalance had only a minimal impact on the odds ratio and confidence interval. Likewise, imputing missing values had very little effect on the results – for instance, the unadjusted results changed to an odds ratio of 0.56 (95% CI 0.29, 1.09), p=0.086. For the three secondary outcomes at 6 months there was also only weak evidence of a (beneficial) effect of the intervention (Table 3), predominantly in anxiety score. Again, imputing missing outcome data had no appreciable effect on these results (data not shown).

**Table 2:** Differences in reported Medically Unexplained Vaginal Discharge (MUVD) at 6 months across groups

	**Intervention**	**Control**	**% Difference (95% CI)**	**OR (95% CI)**	**p-value**
Unadjusted^a^	36/75 (48.0%)	46/73 (63.0%)	-15.0% (-30.8%, 0.1%)	0.54 (0.28, 1.05)	0.067
Adjusted^b^				0.46 (0.22, 0.98)	0.045
Adjusted^c^				0.46 (0.21, 0.98)	0.043

^a^ primary intention-to-treat (ITT) analysis based on the 148 women with known primary outcome status.

^b^ ITT adjusted for women’s work type, husband’s education, VD bothersomeness, wet underpants, reason for VD (n=145).

^c^ ITT adjusted for women’s work type, husband’s education, VD bothersomeness, wet underpants, reason for VD and baseline HSCL-25 Anxiety and Depression scores (n=145).

**Table 3:** Unadjusted and adjusted differences in HSCL-25 Anxiety, HSCL-25 Depression and somatisation scores at 6 months

	**Difference between means**^**a**^**(Intervention minus Control) (95% CI)**	**p-value**	**Adjusted difference**^**b**^**(95% CI)**	**p-value**
HSCL-25 Anxiety	-0.16 (-0.32, 0.01)	0.06	-0.16 (-0.33, 0.02)	0.08
HSCL-25 Depression	-0.09 (-0.24, 0.06)	0.24	-0.10 (-0.27, 0.06)	0.22
Somatisation score	-0.81 (-4.90, 3.27)	0.69	-0.43 (-4.93, 4.07)	0.85

^a^ adjusted for baseline score on the outcome involved.

^b^ adjusted for baseline score, women’s work type, husband’s education, VD bothersomeness, wet underpants, reason for VD and baseline HSCL-25 anxiety and depression scores.

Consenting women in the intervention arm were assigned to one of ten groups (median of 9 women per group; all but two in the range 7-12). Adjusting for clustering by intervention group had very little impact on the primary analysis, with virtually identical results to those in Table 3. From Table 4, there was no evidence of mediating effects for the primary outcome in terms of anxiety, depression or somatisation scores. In respect of adherence, none of those in the control group received any of the intervention prior to the primary outcome being ascertained, while 49/75 (65%) women in the intervention group attended at least six sessions. From the instrumental variables regression analyses, the risk of MUVD was reduced in absolute terms by 2.4 percentage points for each session attended (95% CI -4.9%, 0.0%, p=0.049).

**Table 4:** Assessing the mediating effect of HSCL-25 Anxiety, HSCL-25 Depression and the somatisation scale scores on the primary analysis of MUVD as the outcome

**Possible mediating variable**	**OR**^**a**^**(95% CI)**	**p-value**
Change in HSCL-25 Anxiety score at 6 months	0.57	0.10
	(0.30, 1.12)	
Change in HSCL-25 Depression score at 6 months	0.57	0.10
	(0.29, 1.12)	
Change in somatisation score at 6 months	0.54	0.12
	(0.25, 1.18)	

^a^ OR for MUVD for intervention compared with control adjusting for the possible mediating variable.

Introducing interaction terms between randomization group and age, HSCL-25 Anxiety and Depression scores provided no evidence of differential effects of the intervention on MUVD (p values of 0.83, 0.19 and 0.43 respectively).

## Discussion

### Summary of findings

This is the first community-based randomized trial on MUVD using a group multi-component intervention. There was marginal evidence of a beneficial effect of the intervention in terms of reducing MUVD and anxiety at six months, but with no evidence that the former was mediated by the latter. The confidence intervals rule out any important deleterious effects of the intervention but the precision attained leaves equivocal results as to whether there is a clinically important benefit from the intervention. A reduction of 15 percentage points in the proportion reporting MUVD at 6 months in favour of the intervention is close to the target difference of 20, and the ‘upper’ confidence limit for this difference reaches 30 percentage points. If there were to be a reduction in risk of 2 percentage points per session attended in a population where this condition is highly prevalent then there is the potential for substantial population benefits.

### Limitations and strengths

The logistical difficulties in recruitment meant that randomization was not fully concealed, and this could explain some of the observed disparities across the arms at baseline. There is clear evidence, however, to suggest that these small imbalances did not impact on the results and conclusions. The imprecision in the estimate of effectiveness partly reflects the original target difference, which was large for such an intervention with this degree of intensity and duration. Furthermore, the proportions with MUVD at follow-up were larger (and closer to 50%) than anticipated. Focusing the trial on a population with low to moderate levels of CMD may have limited the extent to which the intervention could reduce psychological distress and the primary MUVD outcome. Most pragmatic trials such as this with usual care as control arm might be affected by a Hawthorne or 'attention' effect. Nonetheless adding an 'attention control group' would have made this trial less pragmatic. Finally we have used self-reported questionnaires rather than psychiatric interviews because the former are likely to be used in the clinics unlike the latter which are long and may require specialist input. This may have introduced some misclassification of cases but most likely similar across arms so that effect sizes were unlikely to be affected by this potential problem.

The study strengths include the approach to recruitment, which was characterized by a wide scale engagement of the community at all stages – planning, implementation and evaluation. Study participants reported high levels of satisfaction and attendance was good [20]. Attrition rates were low for the study context, and from the results of the analyses employing multiple imputations there was very little bias introduced through missing outcome data. Despite the problems encountered with concealment of randomization, the results suggest that the primary analysis could be a reflection of the effectiveness of the intervention.

### Relationship with the existing literature

There is evidence from randomized controlled trials that simple psychological interventions can help to alleviate CMD, in a variety of settings [30-33]. There is also some evidence that interventions designed to increase physical activity can help to alleviate psychological symptoms [34,35]. There have been attempts to investigate the effects of interventions designed to reduce medically unexplained symptoms, but with very limited success [36]. As far as we are aware, the present study is the first to evaluate the effectiveness of a multi-component intervention of this kind in relation to MUVD, especially in a developing country and with attention to potential mediation through CMD.

### Mechanisms of change

The multi-component intervention investigated was intended to reduce anxiety and depressive symptoms, and in turn reduce MUVD. The effects of the intervention on these (secondary) outcomes were marginal, though the fact that results were clearer for anxiety may indicate that MUVD reflects uncertainty about the origins of a presumably physical problem. However, the mediation analyses did not add to understanding the mechanism involved in any change in MUVD. Since the intervention was not designed to focus on somatisation itself, it is less surprising that this neither changed nor contributed any mediating influence.

### Implications of the findings

This study confirms that MUVD is an important public health problem, highly resistant to change – even in the intervention group; nearly half of the women still had MUVD after six months. In settings with limited resources including few trained psychotherapists, as well as long periods of conflict and instability, the use of this potentially low cost, acceptable and easy to run group intervention package is worth considering. While the benefits of this intervention may appear modest, the intervention offers an opportunity for women to enhance their problem-solving skills as well as apply physical relaxation techniques that can help them deal more readily with stress in their lives.

Also it is important to note that due to a portion of women who refused to participate in the study, it is difficult to generalize the results to the larger population of women in Lebanon or in this area since the sample of women who did not participate may have particular characteristics which are reflected in the general population.

Further research is still needed in a variety of contexts, for different populations and preferably involving larger randomized trials of this intervention (or adaptations of it). This would enable smaller but potentially important effect sizes to be estimated with sufficient precision, which is crucial given the public health implications for what is a widespread, costly and cumbersome problem for women in such contexts.

## Conclusion

This study confirms that MUVD is an important public health problem. While the benefits of this intervention may appear modest, the intervention offers an opportunity for women to enhance their problem-solving skills as well as use physical relaxation techniques that can help them deal with stressful in their lives. Further research is needed in a variety of contexts, for different populations and preferably involving larger randomized trials of such an intervention.

## Competing interests

The authors have no competing interests.

## Authors’ contributions

All authors contributed equally to this paper. All authors read and approved the final manuscript.

## Disclosure

The corresponding author has received from all the co-authors disclosure forms for conflict of interest filled out.

## Ethical approval

This study was granted ethical approval by the Institutional Review Board of the American University of Beirut.

## Pre-publication history

The pre-publication history for this paper can be accessed here:

http://www.biomedcentral.com/1471-244X/12/195/prepub
